# Comparison of biomechanical parameters of two Chinese cervical spine rotation manipulations based on motion capture and finite element analysis

**DOI:** 10.3389/fbioe.2023.1195583

**Published:** 2023-07-27

**Authors:** Dongxin Lin, Zaopeng He, Rui Weng, Yuhua Zhu, Zhiwei Lin, Yuping Deng, Yang Yang, Jinchuan Tan, Mian Wang, Yanbin Li, Gang Huang, Guanghao Yu, Daozhang Cai, Xuecheng Huang, Wenhua Huang

**Affiliations:** ^1^ Guangdong Engineering Research Center for Translation of Medical 3D Printing Application, Guangdong Provincial Key Laboratory of Medical Biomechanics, National Key Discipline of Human Anatomy, School of Basic Medical Sciences, Southern Medical University, Guangzhou, China; ^2^ Center for Orthopaedic Surgery, Department of Orthopedics, The Third Affiliated Hospital of Southern Medical University, Guangzhou, China; ^3^ The Third School of Clinical Medicine, Southern Medical University, Guangzhou, China; ^4^ Department of Hand and Foot Surgery and Plastic Surgery, Affiliated Shunde Hospital of Guangzhou Medical University, Foshan, China; ^5^ The Third Affiliated Hospital of Guangzhou University of Chinese Medicine, Guangzhou, China; ^6^ Guangdong Research Institute for Orthopedics and Traumatology of Chinese Medicine, Guangzhou, China; ^7^ Department of Spinal Surgery, Nanfang Hospital, Southern Medical University, Guangzhou, China; ^8^ Orthopedic Center, Affiliated Hospital of Guangdong Medical University, Zhanjiang, China; ^9^ Department of Human Anatomy and Histoembryology, School of Basic Medical Sciences, Guangdong Medical University, Dongguan, China; ^10^ Department of Orthopedics and Traumatology, Integrated Hospital of Traditional Chinese Medicine, Southern Medical University, Guangzhou, China; ^11^ Institute of Biomedical Engineering, Shenzhen Bay Laboratory, Shenzhen, China; ^12^ Mudanjiang Medical University, Mudanjiang, Heilongjiang, China; ^13^ Orthopedic Hospital of Guangdong Province, Academy of Orthopedics, Guangzhou, China; ^14^ Shenzhen Hospital of Guangzhou University of Chinese Medicine (Futian), Shenzhen, China

**Keywords:** motion capture, finite element analysis, cervical rotation manipulation, oblique pulling manipulation, cervical rotation-traction manipulation, biomechanics

## Abstract

**Objective:** The purpose of this study was to obtain the stress-strain of the cervical spine structure during the simulated manipulation of the oblique pulling manipulation and the cervical rotation-traction manipulation in order to compare the mechanical mechanism of the two manipulations.

**Methods:** A motion capture system was used to record the key kinematic parameters of operating the two manipulations. At the same time, a three-dimensional finite element model of the C0-T1 full healthy cervical spine was established, and the key kinematic parameters were loaded onto the finite element model in steps to analyze and simulate the detailed process of the operation of the two manipulations.

**Results:** A detailed finite element model of the whole cervical spine including spinal nerve roots was established, and the validity of this 3D finite element model was verified. During the stepwise simulation of the two cervical spine rotation manipulations to the right, the disc (including the annulus fibrosus and nucleus pulposus) and facet joints stresses and displacements were greater in the oblique pulling manipulation group than in the cervical rotation-traction manipulation group, while the spinal cord and nerve root stresses were greater in the cervical rotation-traction manipulation group than in the oblique pulling manipulation group. The spinal cord and nerve root stresses in the cervical rotation-traction manipulation group were mainly concentrated in the C4/5 and C5/6 segments.

**Conclusion:** The oblique pulling manipulation may be more appropriate for the treatment of cervical spondylotic radiculopathy, while cervical rotation-traction manipulation is more appropriate for the treatment of cervical spondylosis of cervical type. Clinicians should select cervical rotation manipulations for different types of cervical spondylosis according to the patient’s symptoms and needs.

## 1 Introduction

Cervical spine rotation manipulation including cervical rotation manipulation and oblique pulling manipulation is one of the methods recommended by clinical guidelines when no compression symptoms are present and neck pain symptoms are grade I-II and last for ≦3 months (Côté et al., 2016). Cervical spondylotic radiculopathy is caused by unilateral or bilateral spinal nerve root irritation or compression, which manifests as sensory, motor and reflex disorders consistent with the distribution area of spinal nerve roots. There are clinical retrospective and prospective studies and meta-analyses showing that manipulation characterized by high speed and low amplitude cervical rotation thurst can rapidly relieve the radicular symptoms of cervical spondylotic radiculopathy by enlarging the intervertebral foramen volume and releasing the adhesions around the nerve roots and joints to a certain extent (Chang et al., 2022; Childress and Becker, 2016; Groisman et al., 2020; Kuligowski et al., 2021; Romeo et al., 2018; Thoomes, 2016; Zhu et al., 2016). Although cervical spine rotation manipulation is widely used and accepted, the effectiveness of this technique is still controversial in clinical practice. There are also many reports in the literature of adverse reactions to cervical spine rotation manipulation in the treatment of cervical spondylotic radiculopathy, such as aggravation of cervical disc herniation, injury to spinal nerve roots and aortic entrapment, and stroke (Birkett et al., 2020; Kranenburg et al., 2017; Moser et al., 2019; Swait and Finch, 2017). This may be due to the fact that the biomechanical mechanism of the manipulation is still unclear.

The main research methods for cervical spine biomechanics are *in vitro* models and *in vivo* models. The cadaver specimens has good human representation and can provide practical and reliable model support for cervical spine biomechanical studies, but human specimens are difficult to obtain due to the constraints of medical ethics and traditional morality. Human *in vivo* models are limited in their application due to the strong restrictions of medical ethics (Cho et al., 2022; Kauther et al., 2014). In recent years, with the development of science and technology, the emergence of computer simulation technology and finite element analysis methods has provided brand new research methods and techniques for cervical spine biomechanics research (Purushothaman and Yoganandan, 2022; Rong et al., 2017). The application of finite element analysis methods in cervical spine biomechanics refers to the application of imaging and mathematical methods to restore the structural morphology of the cervical spine, define the loading boundary conditions and additional material properties. And the effect on the mechanical properties of the cervical spine structure is observed by changing the parameters and compared with the mechanical properties of the cervical spine in the physiological state, so as to explain the effect of the pathological process on the mechanical properties of the cervical spine. Finite element analysis allows for more accurate simulation of subtle morphological changes in the external and internal structures of tissues in response to mechanical forces and can be reused, greatly reducing research costs. The advent of 3D finite element models of cervical discs provides a new approach to the mechanistic study and therapeutic evaluation of spine-related diseases (Cao et al., 2021; Frantsuzov et al., 2023; Komeili et al., 2021; Rycman et al., 2021; Vedantam et al., 2023; Xu et al., 2022; Yang et al., 2022).

Therefore, the purpose of this study is to simulate the whole cervical spine structure, and the use of motion capture combined with 3D finite element model can further reveal the mechanical state of these two manipulations applied to the cervical spine, the changes of force conduction within the cervical spine and the changes of the spatial structure of the cervical spine caused by the force, which is beneficial to the study of the mechanism of the mechanical action of the manipulation.

## 2 Materials and methods

### 2.1 Acquisition of kinematic parameters for two manipulations

**FIGURE 1:**
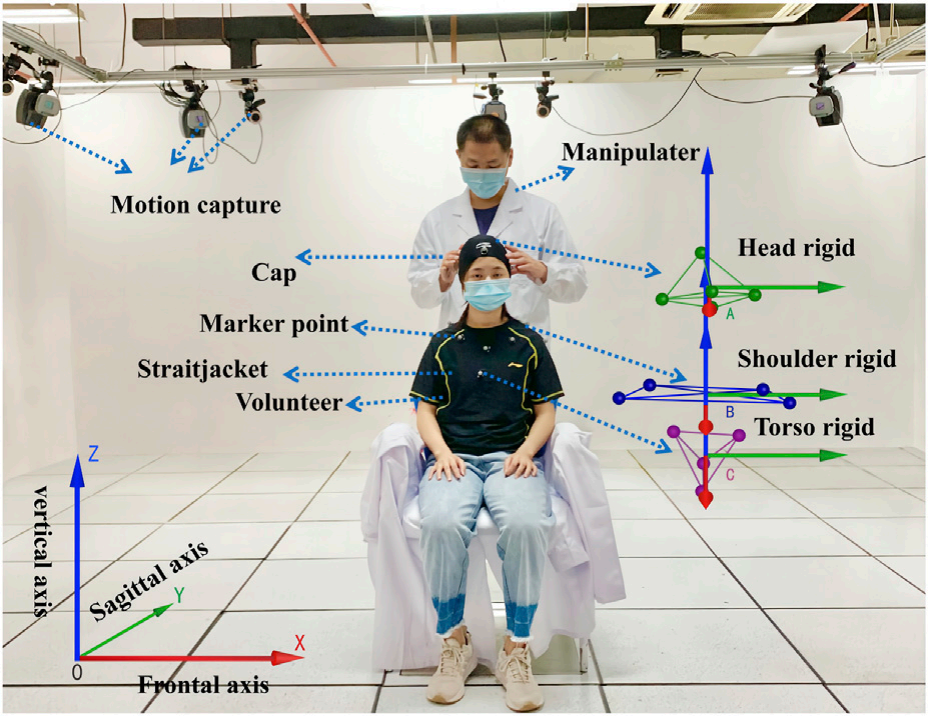
Mechanical parameters of the cervical rotation manipulation obtained with motion capture monitoring.

A total of 48 volunteers (20 women and 28 men) aged from 24 to 30 years old, who had no pathological changes after X-ray examination, were selected. They were randomly divided into the group of the oblique pulling manipulation and the group of the rotation–traction manipulation. A total of 24 subjects were in each group. The motion capture system and Visual 3D software were used to obtain and analyse kinematic parameters. After volunteers put on straitjackets and caps, 13 special marker points were placed in the head and trunk to establish three-dimensional models. The specific positions were as follows (as shown in Figure 1): five marker points were on the head (one point each on the bilateral temporal regions, one point on the forehead, one point on the vertex, and one point on the occipital region), four points on the shoulder and neck (one point each on the bilateral acromions and one point each on the midline bilateral clavicles), and a four points on the trunk (one point each on the bilateral pectoralis major muscles, one point under the xiphoid, and one point on the upper abdomen). The kinematic parameters of two cervical rotation manipulations have been obtained in our preliminary study (Huang et al., 2021). The schematic diagram is shown in Figure 1.

### 2.2 FE modeling

A healthy Chinese male volunteer (age 28 years, height 172 cm, body weight 65 kg) was recruited for this study. The subject’s skull and vertical spine were scanned using a 64-row spiral CT scanner (SOMATOM Definition AS +, Siemens, Germany). A total of 289 CT slices were acquired, layer thickness 1.25 mm, interval 0.625 mm, field of view: 350 mm. The study was reviewed by the Medical Ethics Committee of Southern Hospital of Southern Medical University and volunteers signed an informed consent form (permission number:1,date:2022-01-02).

**FIGURE 2:**
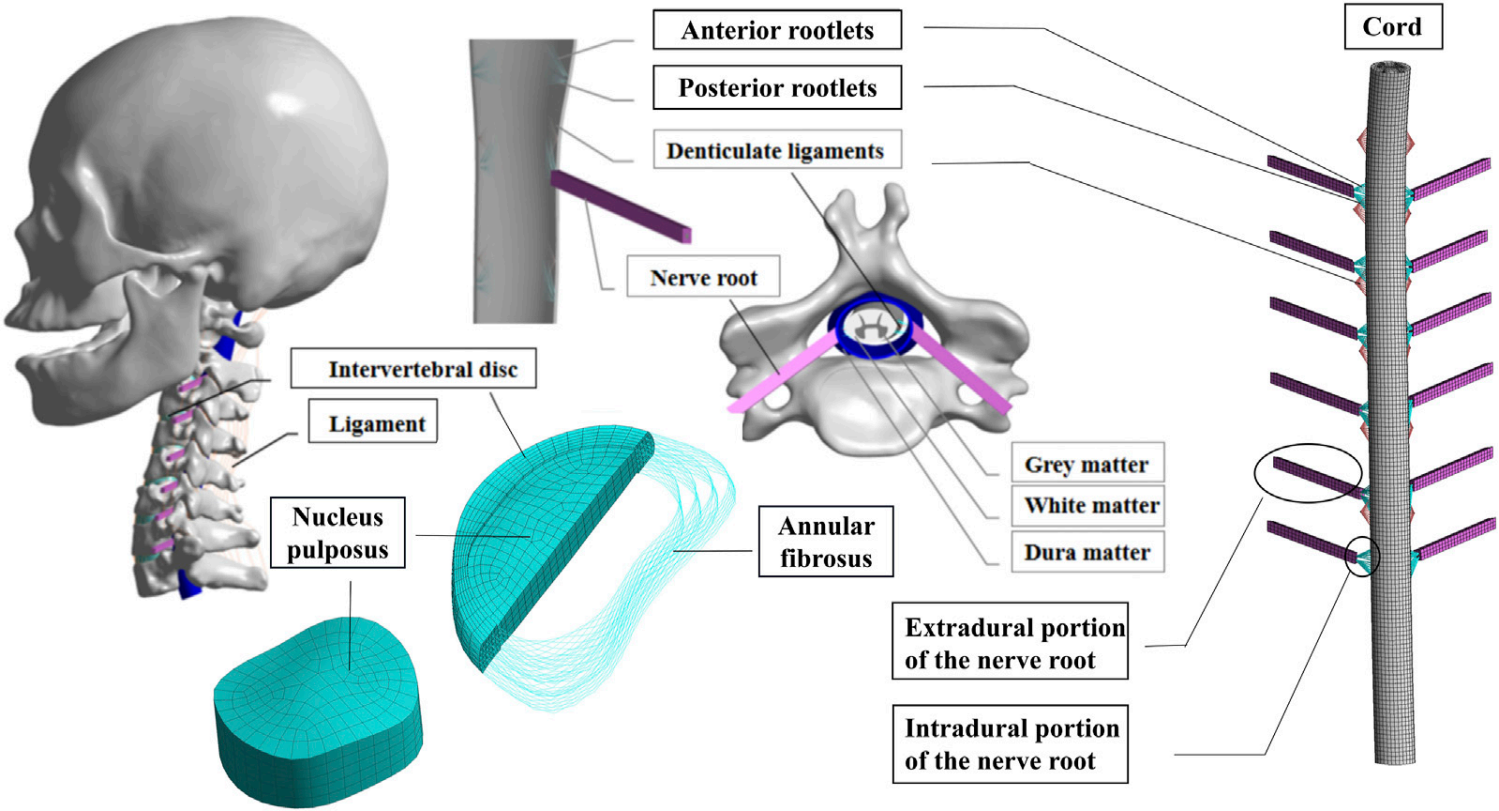
Full cervical spine finite element model, including detailed structures of the intervertebral discs, facet joints, spinal cord and nerve roots.

A detailed three-dimensional (3D) nonlinear finite element model of the complete Skull and C1-T1 spine was created from cross-sectional CT images. The DICOM format image file was read using the medical 3D reconstruction software Mimics 20.0 software (Belgium Materialise company). The bone tissue was segmented by different thresholds of bone tissue and soft tissue, and then the cranial and C0-T1 vertebrae information was extracted by threshold segmentation, erasure and filling functions. The vertebral bone connecting parts of each segment were manually separated and cavity filling was performed. Finally, the edited mask is 3D transformed to generate the cranial and C0-T1 vertebral facet network models. The models were exported as point cloud format files from Mimics 20.0 software and imported into Geomagic Studio 12.0 (USA Geomagic company) reverse engineering software. Smoothly process the vertebral models and use the surface function to generate geometric solids from the respective vertebral models and export them as stp files. The cervical C1-T1 vertebrae model was then imported into Solidwords 2015 (France, Dassault Company) to compose the assembly, and the cortical bone, fibrous ring, nucleus pulposus, spinal cord, nerve roots, cartilage endplates, synovial articular cartilage and ligaments were modeled on the basis of the C1-T1 vertebrae contour. The occipital bone is connected to C1 through the atlanto-occipital joint. The vertebral body consists of entities such as cortical and cancellous bones and upper and lower cartilage endplates (Mo et al., 2014; Mo et al., 2015). The thickness of the cartilage layer of the facet joint was assumed to be 0.2 mm. The intervertebral disc is a continuous structure containing the nucleus pulposus and the fibrous ring. The nucleus pulposus is located in the central posterior position of the disc and accounts for 43% of the disc volume, while the annulus fibrosus accounts for 57% of the disc volume (Denozière and Ku, 2006). The annulus fibrosus is further divided into the annulus fibrosus matrix and the annulus fibrosus fibers, where the volume of fibers of the annulus fibrosus accounts for about 19% of the volume of the annulus fibrosus (Kim et al., 2013). The parallel fiber structures are embedded in the annulus fibrosus matrix, and each layer of the matrix contains two layers of intersecting fibers, with the fibers crossing at an angle of about 15–45° to the horizontal plane. In the radial direction, four double cross-linked fiber layers are defined, which are bounded by a ring matrix and two end plates. In addition, the elastic strength of these fibers decreases proportionally from the outermost layer (550 MPa) to the innermost layer (358 MPa). The articular surfaces of facet joint were modeled using surface-to-surface contact elements combined with a penalty algorithm with a normal contact stiffness of 200 N/mm and a friction coefficient of 0. The thickness of the cartilage layer of the facet joint was assumed to be 0.2 mm. The initial gap between the cartilage layers is assumed to be 0.5 mm. The cartilage was assumed to be isotropic linear elastic with a Young’s modulus of 35 MPa and a Poisson’s ratio of 0.4 (Schmidt et al., 2012; Shirazi-Adl, 1991). The ligaments of the 3D finite element model include anterior atlanto-occipital membrane (AAOM), posterior atlanto-occipital membrane (PAOM), cruciate ligamentum vertical portion (CLV), alar ligament (AP), apical ligament (AL), membranae tectoria (TM), the anterior longitudinal ligament (ALL), posterior longitudinal ligament (PLL), capsular ligament (CL), flaval ligament (FL), interspinous ligament (ISL), supraspinous ligament (SSL), and transverse ligament (TL)with the suggested insertion site (Lafage et al., 2008). The FE model of the spinal cord consists of white matter, gray matter, dura mater, cerebrospinal fluid, epidural and intradural nerve roots, and Denticulate ligaments (DLS). The spinal cord is free at the upper end, fixed as the lower endplate of the C7 vertebra at the lower end. In the finite element model, a frictionless contact between the spinal cord and nerve roots and the vertebrae is set up, which is a nonlinear contact that can produce sliding in both the tangential and normal directions of the contact surface. If the vertebrae touch the spinal cord during anterior and posterior flexion and extension or lateral flexion or rotation, the contact takes effect immediately and the vertebrae compress the spinal cord, generating sliding in the tangential direction while interacting with each other in the normal direction (Khuyagbaatar et al., 2016). The geometry of the cervical medulla was established by quantitative measurements of the human spinal cord (Khuyagbaatar et al., 2016). The dura was placed at a distance of approximately 2.5 mm from the spinal cord, as the cerebrospinal fluid layer in the human cervical spine was experimentally shown to occur at a distance of 1.5–4.0 mm (Kameyama et al., 1996). Nerve roots were modeled based on a microsurgical anatomical study (Alleyne et al., 1998), where the nerve roots consisted of an extradural and intradural portion. The extradural nerve root was simplified into two different materials of outer layer wrapped around the inner layer of the column-shaped body, where the outer layer adopts an elastic modulus of 80 MPa, poisson’s ratio of 0.49, and the inner layer of 1.3 MPa, poisson’s ratio of 0.3. The solid186 cell was used for its hexahedral meshing with a mesh size of 1 mm. The inner and outer layers of the cell are connected with a common node. The nerve root was connected to the dura mater using bound contact connection. Meanwhile, through the apdl command flow insertion function of the ansys workbench software, the command to mesh the intradural nerve root before and after using the spring unit combin39 and assigning the characteristic nonlinear force-displacement curve that can be stretched only is prepared, and the command is executed when waiting for the subsequent calculation (Singh et al., 2006). The DLS was modeled at each spinal level as 22 triangular extensions that connect laterally from the spinal cord to the dura mater (Ceylan et al., 2012). Fluid elements were modeled as Eulerian elements, which are arbitrary cubic sets of elements that completely encompass the fluid material region during the analysis. The volume between the dural sheath and the cord was filled with Eulerian material and the interaction between the fluid material and the solid was studied by Eulerian-Lagrangian analysis using Ansys Workbench 18.0 (USA, Ansys Company) (Bloomfield et al., 1998). The dura and epidural nerve roots were assumed to represent a single tangential modulus from the experimental study (Bloomfield et al., 1998). The intradural nerve roots and DLS are represented by purely tensile spring elements with nonlinear force-strain relationships. The material properties of cerebrospinal fluid were demonstrated using Newtonian fluids characterized by the viscosity of the cerebrospinal fluid. The above models were imported in the finite element analysis software Ansys Workbench 18.0 for assembly, material property assignment, interrelationship definition, and meshing, with the fiber ring fibers and ligaments defined as subject to tensile forces only without pressure or shear (as shown in Figure 2) (Khuyagbaatar et al., 2017). The material properties were defined as linear, homogeneous, and isotropic, and the constructed model was assigned with the material properties shown in Table 1.

**TABLE 1:** Material properties of the full cervical spine finite element model.

Component	Element type	Young’s modulus (MPa)	Poisson’s ratio	Cross-sectional area (mm^2^)
Vertebral cortical bone	Tetra element	12,000	0.29	-
Vertebral cancellous bone	Tetra element	450	0.29	-
Posterior vertebral structures	Tetra element	3,500	0.29	-
Endplate	Tetra element	500	0.4	-
Nucleus pulposus	Tetrahedron	1	0.49	-
Annulus (ground substance)	Tetrahedron	3.4	0.4	-
Annulus fiber	Tension-only elasticity	358–550	0.30	-
AAOM	Tension rod unit	10	0.3	6.0
PAOM	Tension rod unit	1.5	0.3	5.0
CLV	Tension rod unit	10	0.3	5.0
AP	Tension rod unit	10	0.3	5.0
AL	Tension rod unit	5	0.3	22.0
TM	Tension rod unit	10	0.3	6.0
ALL	Tension rod unit	10	0.3	6.0
PLL	Tension rod unit	10	0.3	5.0
LF	Tension rod unit	1.5	0.3	5.0
ISL	Tension rod unit	1.5	0.3	10
TL	Tension rod unit	1.5	0.3	10
SSL	Tension rod unit	1.5	0.3	5
CL	Tension rod unit	10	0.3	46
White matter	Tetra element	0.004	0.499	-
Gray matter	Hex element	0.0041	0.499	-
Dura mater	Tetra element	80	0.49	-

### 2.3 Model validation

**FIGURE 3:**
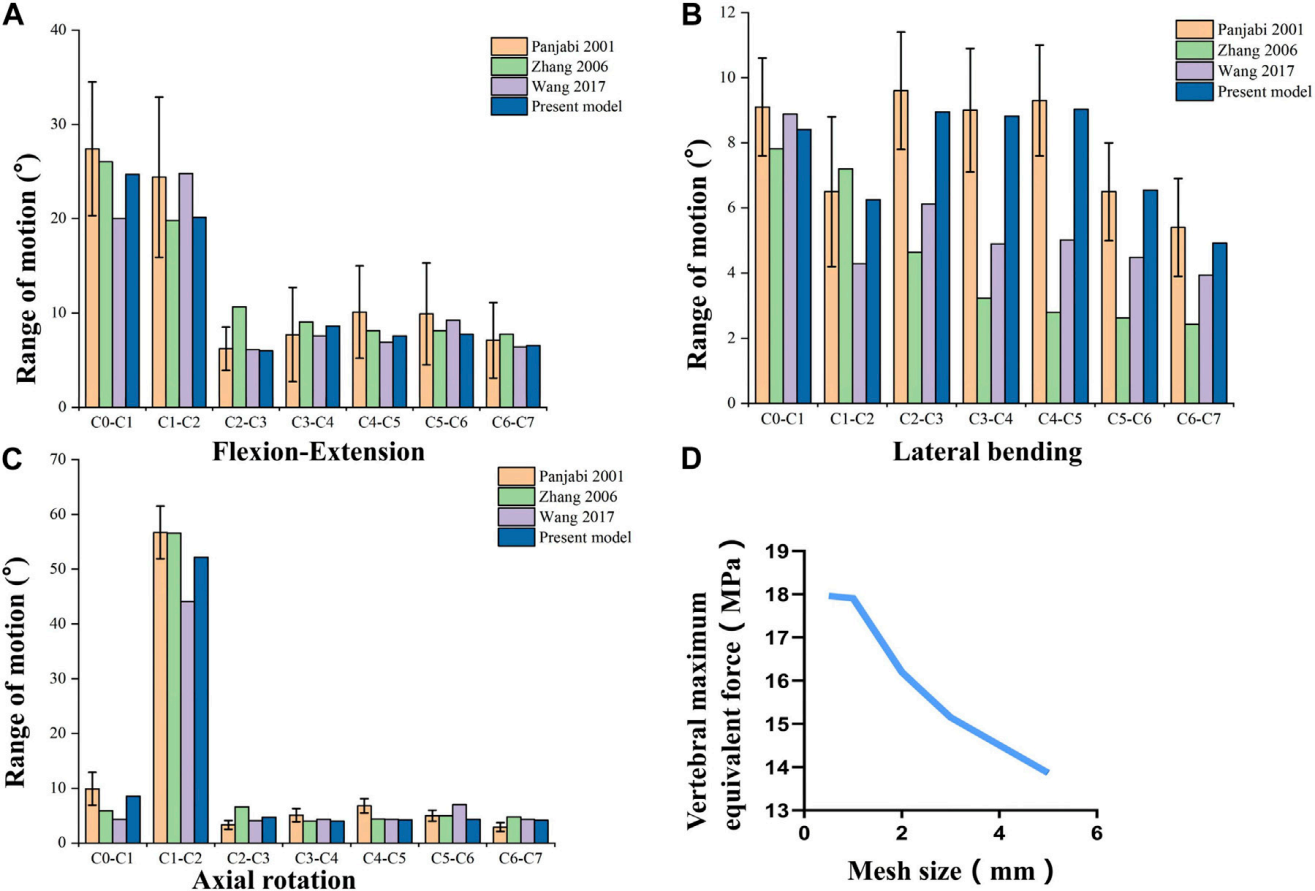
Validation of the Full cervical spine finite element model. **(A)** Flexion-Extension range of motion. **(B)** Lateral bending range of motion. **(C)** Axail rotation range of motion. **(D)** Mesh convergence test.

To verify the finite element model of C1-C7, the lower end plate of C7 was fixed and then applied a pure moment of 1.5 Nm at the level of C0 (Lee et al., 2011). Based on previous studies, the range of motion (ROM) was compared with the results of the experimental analysis study by Panjabi et al. (Panjabi et al., 2001) and other experiments (Wang et al., 2017; Zhang et al., 2006) under the respective loading conditions to assess the validity of the cervical spine finite element model (As shown in Figure 3). In general, the validation results will be accepted as good agreement when the calculated ROM values are within ±1 standard deviation of the mean of the *in vitro* measurements. Five mesh sizes of 0.5mm/1mm/2/mm/3mm/5 mm were used as mesh convergence tests, and it was found that when the mesh was 0.5 mm and 1mm, the difference in tissue stresses such as vertebral body and intervertebral disc was within 5%, while the difference in time was several times (as shown in Figure 3D). Combining the computational accuracy and efficiency, 1 mm was chosen as the mesh size for this study, resulting in 446,263 nodes and 226,402 cells.

### 2.4 Manipulation stimulation

**FIGURE 4:**
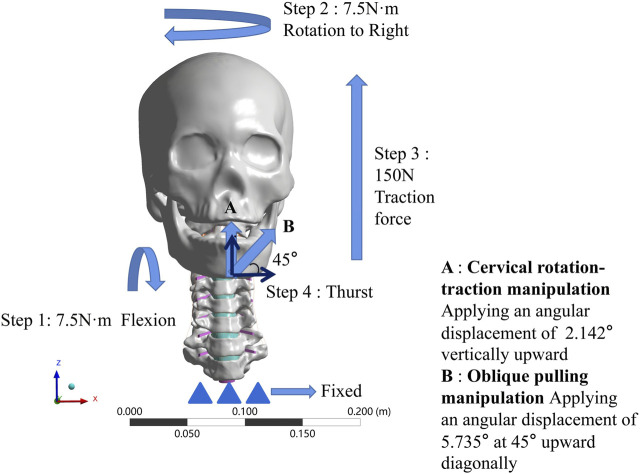
Boundary condition loading diagram for two manipulations. **(A)** The cervical rotation-traction manipulation. **(B)** The oblique pulling manipulation.

Then we loaded the key kinematic parameters of the two cervical rotation manipulations previously obtained by monitoring the volunteers using motion capture equipment onto the validated 3D finite element model in a stepwise manner (As shown in Figure 4). The *X*-axis represents the frontal axis, the *Y*-axis represents the sagittal axis, and the *Z*-axis represents the vertical axis. After fixing the lower endplate of T1, the sequence of mechanical loading steps for the simulated CSM were as follows.

#### 2.4.1 The cervical rotation-traction manipulation

(1) Apply 7.5N·m bending angular displacement to the mandible around the *X*-axis direction, simulate forward flexion.
(2) Keep 7.5N·m bending angular displacement around the *X*-axis direction, while in the same time 7.5N·m bending angular displacement around the *Z*-direction, simulate the rotation to the right.
(3) On the basis of keeping steps 1 and 2, then apply 150N in the *Z* direction for 5s to simulate a vertical upward force applied to the fixed mandible.
(4) On the basis of the first three steps, rotate the mandible around the *X*-axis by 2.142° for 0.25s to simulate the wrenching process of the cervical rotation-traction manipulation.

#### 2.4.2 The oblique pulling manipulation

(1) Apply 7.5N·m bending angular displacement to the mandible around the *X*-axis direction, simulate forward flexion.
(2) Keep 7.5N·m bending angular displacement around the *X*-axis direction, while in the same time 7.5N·m bending angular displacement around the *Z*-direction, simulate the rotation to the right.
(3) On the basis of keeping steps 1 and 2, then apply 150N in the *Z* direction for 5s to simulate a vertical upward force applied to the fixed mandible.
(4) Establish a local coordinate system. Let the new *X*-axis be 45° from both *X*-axis and *Z*-axis under the original whole, and then apply an angular displacement of 5.735° along the new *X*-axis. Simulate the thurst process of the oblique pulling manipulation.

The boundary conditions that we initially wanted to use in this study were the data obtained from the pre-motion capture at each step. However, in the actual action, flexion can reach more than 15° and the rotation can reach more than 60°, but the finite element model simply cannot converge if it is loaded as it is. So we used the moments from the validated finite element model to simulate flexion and rotation, the upward force at step 3 and the thurst angular displacements at the last step of thursting were those obtained by motion capture.

## 3 Results

**FIGURE 5:**
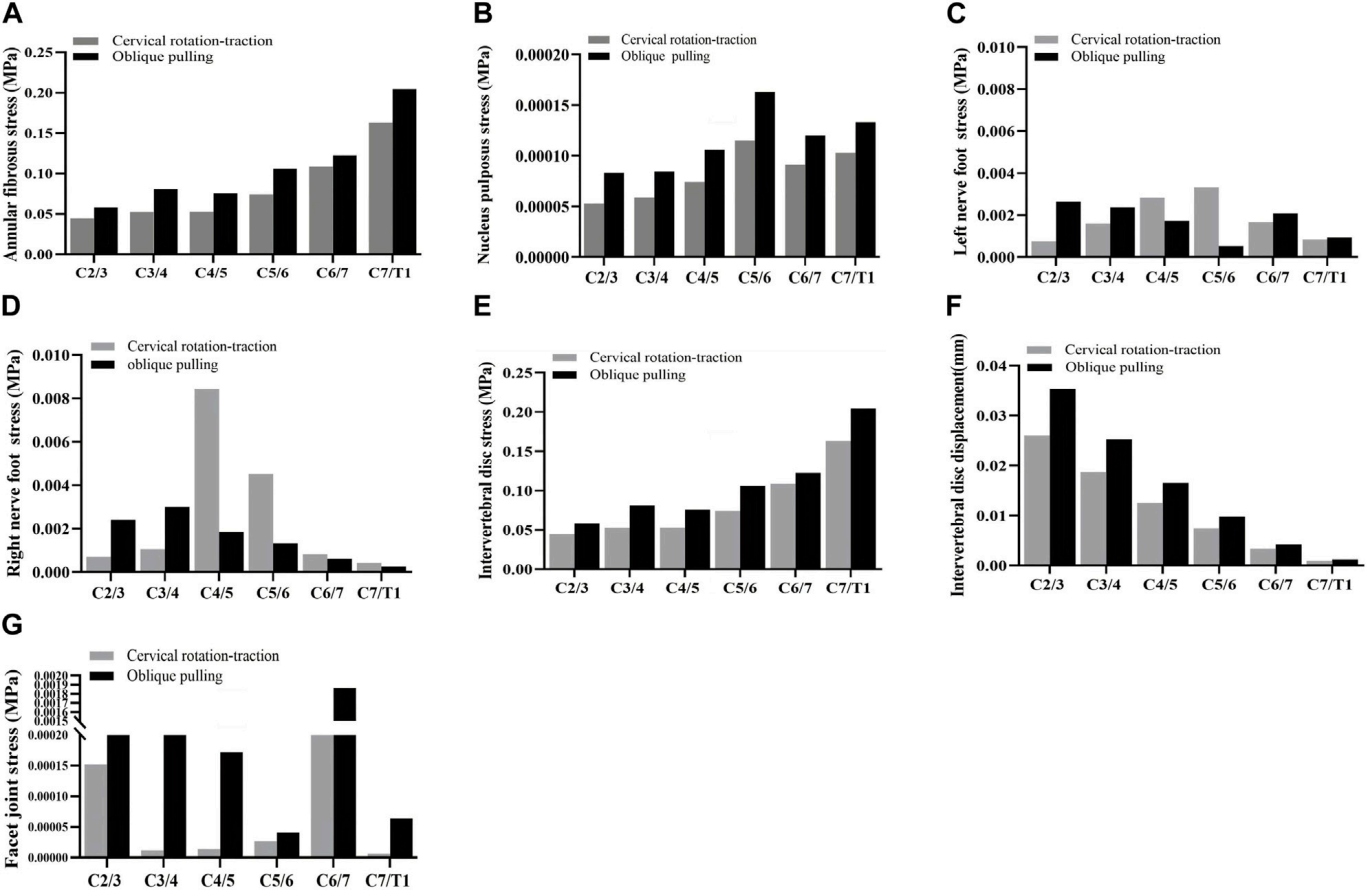
Stress-strain statistics of each structure. **(A)** Von-Mises Stress of the annular fibrosus. **(B)** Von-Mises Stress of the nucleus pulposus. **(C)** Von-Mises Stress of the left nerve foot. **(D)** Von-Mises Stress of the right nerve foot. **(E)** Von-Mises Stress of the intervertebral disc. **(F)** Displacement of the intervertebral disc. **(G)** Von-Mises Stress of the facet joint.

**FIGURE 6:**
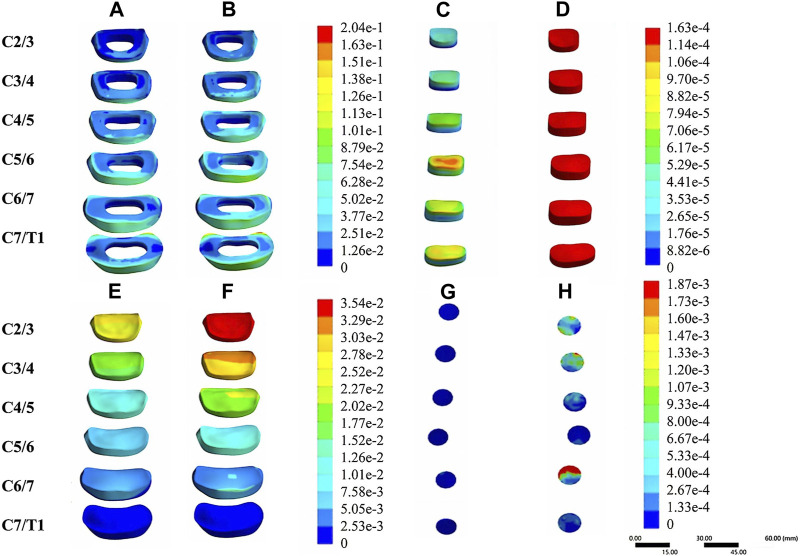
Stress-strain patterns of the intervertebral disc and facet joints under two manipulation simulations. **(A)** The von-mises stress of the annular fibrosus in the cervical rotation-traction manipulation. **(B)** The von-mises stress of the annular fibrosus in the oblique pulling manipulation. **(C)** The von-mises stress of the nucleus pulposus in the cervical rotation-traction manipulation. **(D)** The von-mises stress of the nucleus pulposus in the oblique pulling manipulation. **(E)** The von-mises stress of the intervertebral disc in the cervical rotation-traction manipulation. **(F)** The von-mises stress of the intervertebral disc in the oblique pulling manipulation. **(G)** The von-mises Stress of the facet joint in the cervical rotation-traction manipulation. **(H)** The von-mises stress of the facet joint in the oblique pulling manipulation.

**FIGURE 7:**
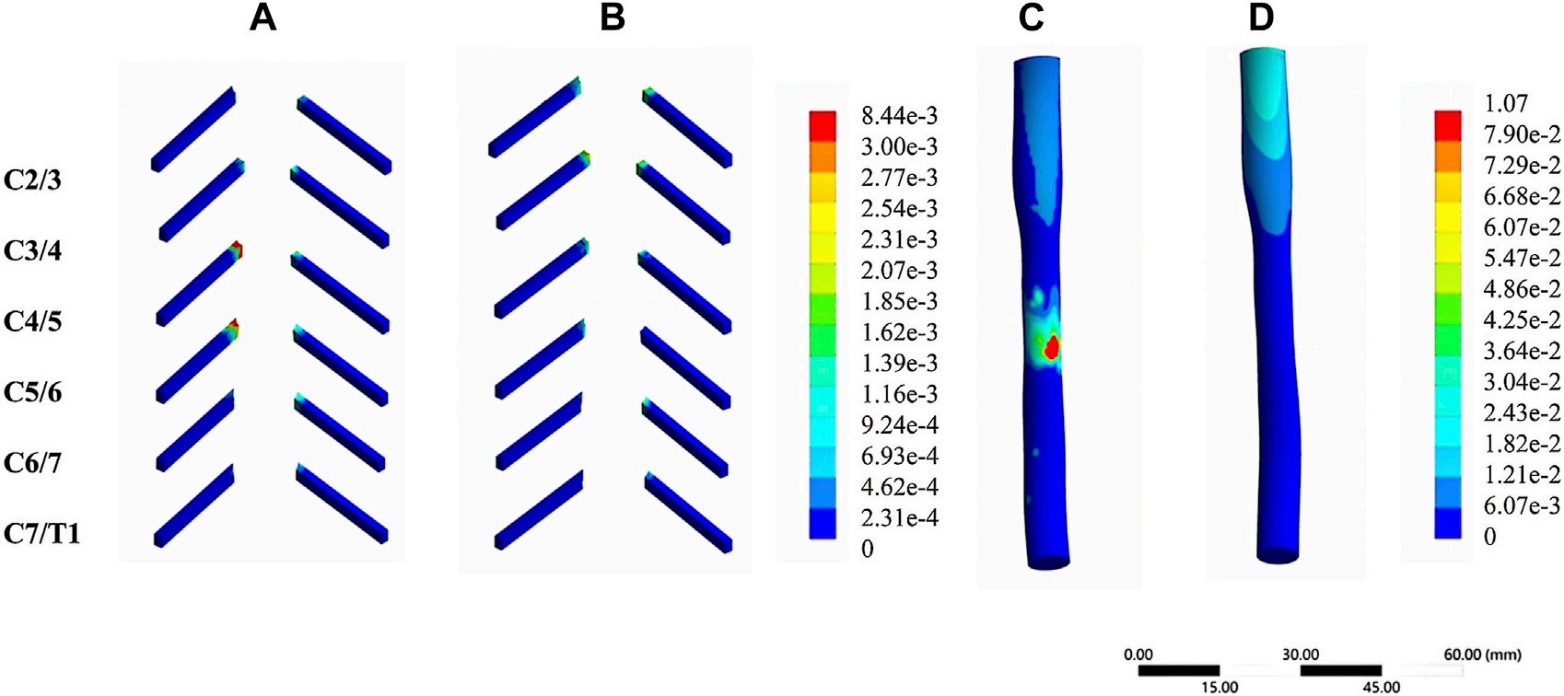
Stress-strain patterns of the spinal cord and nerve roots under two manipulation simulations. **(A)** The von-mises stress of the nerve roots in the cervical rotation-traction manipulation. **(B)** The von-mises stress of the nerve roots in the oblique pulling manipulation. **(C)** The von-mises stress of the spinal cord in the cervical rotation-traction manipulation. **(D)** The von-mises stress of the spinal cord in the oblique pulling manipulation.

In the validation part of the model, this model was compared with cadaveric experiments and finite element experiments with a similar loading range, and there was a relatively good agreement between our experimental data and the reference data. During the stepwise simulation of the cervical rotation manipulation, the stresses in the annular fibrosus from C2/3 to C7/T1 during the oblique pulling manipulation were greater than those of the cervical rotation-traction manipulation. In addition, stresses of annular fibrosus increased gradually with increasing segment for both manipulations of thrusting (As shown in Figure 5A). The stress in the nucleus pulposus from C2/3 to C7/T1 in the oblique pulling manipulation was greater than that in the cervical rotation-traction manipulation, and the maximum stress in the nucleus pulposus for both manipulations occurred in the C5/6 segment (As shown in Figure 5B). Both manipulations simulated in this study were thursted to the right side. For the nerve roots, the overall stress on the nerve roots on both the left and right sides of the cervical rotation-traction manipulation is greater than that of the oblique pulling manipulation (As shown in Figures 5C, D). The spinal stress in the cervical rotation-traction manipulation group was also greater than that in oblique pulling manipulation group. The spinal cord and nerve root stresses of the cervical rotation-traction manipulation are concentrated in the C4/5 and C5/6 segments. The overall stress and displacement of the disc from C1/2 to C7/T1 during the oblique pulling manipulation were greater than those of the cervical rotation-traction manipulation, but the stress gradually increased with segment and the displacement gradually decreased with segment (As shown in Figures 5E, F). The stresses on the facet joints were free on the right side during the thursting process to the right, and there was no stress. As for the stresses in the left facet joints, oblique pulling manipulation group was greater than the cervical rotation-traction manipulation in every segment (As shown in Figure 5G). The stress pattern (Figure 6) shows that the fibrous ring, nucleus pulposus, and facet joint stresses are greater in the oblique pulling manipulation group than in the cervical rotation-traction manipulation. When thursting to the right, overall, the spinal cord and nerve root stresses on both sides were greater in the cervical rotation-traction manipulation than in the oblique pulling manipulation as shown in the Figure 7.

## 4 Discussion

In this study, the kinematic data obtained from the motion capture were loaded onto a finite element model as boundary conditions to simulate two non-point rotational manipulations, and the stress-strain of the structures of the cervical spine during the thursting of the two manipulations was obtained to analyze the biomechanical mechanism of the two manipulations. The cervical spine non-fixed rotation technique has no fixed point on the cervical spine, and thursts the patient’s head to transmit force to the cervical spine to achieve the therapeutic effect. Both the oblique pulling manipulation and cervical rotation-traction manipulation are non-point rotational manipulations. The non-point rotational manipulations of treatment achieves a therapeutic effect by eliminating facet joint disorders, eliminating abnormal concentrated stresses and correcting force lines so that the imbalance of the cervical spine can be improved (Lin et al., 2012). However, inappropriate thurst forces can directly cause microdamage to the structures surrounding the cervical spine, affecting the physiological homeostasis of the relevant tissues and cells and their functions, leading to disc degeneration and forming a vicious circle. Previously, we obtained kinematic parameters for operating two cervical spine rotation manipulations under motion capture monitoring and compared parameters such as angular displacement of the thursting of the two manipulations. However, motion capture can not quantify the stress-strain of the internal structures of the cervical spine (spinal nerve roots, intervertebral discs, facet joints, etc.). In order to analyze the biomechanical mechanisms of action of the manipulations, we loaded the kinematic parameters obtained from motion capture as boundary conditions onto the 3D finite element model in steps. By cross-applying finite element analysis, motion capture and mechanical measurement techniques, digital simulation of the manipulative treatment process can be realized to make up for the shortcomings of traditional research. In this way, the biomechanical mechanisms of action of the two cervical spine rotation maneuvers in the treatment of cervical spondylotic radiculopathy were compared.

### 4.1 The stress and displacement of the intervertebral disc and facet joints are greater with the oblique pulling manipulation than with the cervical rotation-traction manipulation

Under normal conditions, the nucleus pulposus converts the stresses into pressure on the endplate and tension on the fiber ring by deformation, thus dispersing the stresses. The greater the stress in the nucleus pulposus, the more pronounced the tendency to expand outward through deformation, and the greater the tension on the fibrous ring (Vergroesen et al., 2015). Increased shear stress in the annulus fibrous indicates an increased risk of annulus fibrous tearing (Farshad-Amacker et al., 2014). When there is a tear in the annulus fibrosus, the nucleus pulposus moves along the torn fissure in the annulus fibrosus, resulting in a decrease in intramedullary pressure and disc height (Sharma et al., 2009). At this time, the intervertebral disc stress situation is changed, so that the stress is mainly concentrated in the annulus fibrosus, which is less tolerant to stress, and under the stimulation of larger stress or repeated stress, it will aggravate further damage to the annulus fibrosus, and even cause the nucleus pulposus to protrude and compress the nerve root, causing radicular pain. Both manipulations are done in a forward-flexed position, and rotation to the healthy side can produce a greater displacement of the affected disc to the front, which is conducive to the release of nerve root adhesions and decompression. Although the oblique pulling manipulation produced greater displacement of the disc, it also produced greater stress, suggesting a higher risk of injury to the disc from the manipulation. The facet joints play an important role in sharing the disc load and limiting axial rotation. When the spine does rotational movements, the facet joints on one side separate from each other and squeeze each other, on the other side, acting as a block (Huang et al., 2018; Weng et al., 2022). If the rotation is too large, it may increase the blocking stress on the small joints and weaken the ability of the small joints to limit intervertebral rotation, and axial rotation may damage the annulus fibrosus and make the disc more likely to protrude (Huang et al., 2020). In summary, we concluded that the risk of injury to the intervertebral disc and facet joints is higher with the oblique pulling manipulation compared with the cervical rotation-traction manipulation. This is consistent with our previous findings, in which the angular displacement amplitude of the two cervical spine rotational manipulations was measured by a motion capture system, resulting in a greater thurst amplitude for the oblique pulling manipulation than for the cervical rotation-traction manipulation. The greater amplitude of thurst, the greater stress and displacement of the disc and facet joints.

### 4.2 The stress on the spinal cord and nerve roots is greater with the cervical rotation-traction manipulation than with the oblique pulling manipulation

We also found an interesting phenomenon: the spinal cord and nerve root stresses in the cervical rotation-traction manipulation group were concentrated in C5. This can probably be attributed to the anatomical characteristics of the C5 nerve root. Sakaura et al. (2003) and Kim et al. (2014) found that C4 and 5 segments are more anteriorly convex than other segments, so C5 nerve roots are shorter and less free than other segments, and they are easily stretched during the thurst process, resulting in higher tension. The physiological curvature of the normal human cervical spine is a forward C-shape, and the point of the physiological curvature is located at C4 or C5, which is also the “nerve tether theory” that C5 nerve root paralysis is likely to occur after vertebroplasty. The cervical rotation-traction manipulation is mainly lifting and pulling, which may aggravate the nerve root compression and increase the tension of the nerve root, causing a series of symptoms in the innervated area of the nerve root being compressed. In contrast, oblique pulling manipulation has a larger thursting range and is mainly rotational, which may help to release the adhesions of the nerve roots and relieve the compression.

### 4.3 Analysis of the biomechanical mechanisms of the two manipulations

The non-fixed cervical spine rotation technique acts on the head through the compound moment of forward flexion plus rotation and instantaneous thursting. The key to its operation is to slowly rotate the cervical spine to the maximum angle while pulling it upward to reach elastic fixation, and then instantly and gently force fully rotate it, thus thursting each segment of the cervical spine from top to bottom. Elastic fixation means that after the patient is actively rotated to the limit, the operator then helps the patient to passively rotate to the limit to achieve an elastic fixed position. Cervical spondylotic radiculopathy is caused by unilateral or bilateral spinal nerve root stimulation or compression, and the patient mainly produces radiculopathic symptoms, so the main treatment is to relieve nerve root compression and reduce nerve root inflammation. The oblique pulling manipulation is more suitable for the treatment of radiculopathy because it allows greater displacement of the intervertebral disc forward, which is more conducive to relieving nerve root compression. Cervical spondylosis of cervical type is mainly due to degeneration of the nucleus pulposus (NPs) and increased pressure in the NPs, and patients mainly have axial neck pain rather than radicular symptoms. The main steps of the cervical rotation-traction manipulation include extraction and traction, which helps to reduce the pressure on the nucleus pulposus and relieve axial neck pain. Although there is a risk of C4 and C5 nerve root pulling, cervical spondylosis of cervical type does not have radicular symptoms, so the cervical rotation-traction manipulation is more suitable for the treatment of cervical spondylosis of cervical type.

Meanwhile, we compared the obtained stresses and strains in each segment of the disc with the literature. Casaroli et al. (2017a) reported that in the human disc, the stresses in the disc were up to 0.44 MPa in lateral bending, 0.57 MPa in axial rotation, 0.62 MPa in posterior extension and 0.71 MPa in anterior Casaroli et al. (2017b) concluded that an axial stress of 12 MPa would result in disc failure, i.e., yield stress of the disc, while an axial stress of 4 MPa would not produce any damage. Our finite element model obtained disc stress intervals between 0.023-0.064 MPa and 0.069–0.267 MPa for the spin-lift and tilt-plate maneuvers, respectively, which do not reach the threshold of disc damage, and although the stress on the disc for the tilt-plate maneuver is greater than that for the spin-lift maneuver, neither exceeds the threshold of damage and both are safer cervical rotation Both are safe cervical rotation techniques. We also compared with the effective strain threshold for fibrous annulus disruption (0.4–0.6) reported by Werbner et al. (2017), and both maneuvers were below the injury threshold and did not produce AF failure.

### 4.4 Limitation

The present study still has some limitations. First of all, define spine material properties as homogeneous, linear and isotropic, with some differences from the real human body. However, there were two reasons for considering the use of linear materials at that time. One was that we thought that the difference between linear and nonlinear was smaller in the range of mechanical activities. The other point is because the calculation is much more difficult after using nonlinearity, and sometimes even non-convergence occurs. Secondly, A 3D finite element model of a healthy volunteer was used as the study subject, which failed to simulate the intervertebral discs of CSR (Cervical Spondylotic Radiculopathy) patients. But this study was able to clarify the effects of these two manipulations on the cervical disc, facet joint, nerve roots and spinal cord. Future studies will model different degrees of cervical disc degeneration to simulate the key steps of cervical rotational manipulation for cervical spondylotic radiculopathy and to explore the biomechanical mechanisms of action. Finally, the simulations were performed under idealized conditions without considering muscle forces and without including some ligaments of the neck. And the simulation of the manipulation is not fully consistent with the motion capture. Regarding the fact that the first two steps of the simulation were not based on motion capture data, the explanation is that both Chinese cervical spine manipulations belong to the same class of cervical rotation manipulations, and the first three steps are the same, so the main concern is the biomechanical effect of the last step of manipulation on each structure. Our motion capture simulation technique uses healthy adults and uses normal data to speculate on potential risky injuries. The point we are trying to make is that in a normal cervical spine, which is already more prone to injury, the potential risk is even greater in the patient. And this study cannot be used as a clinical indicator, but only as a predictor of the potential risks that may arise from different manipulations. In the future study, we will use motion capture to obtain two cervical rotation manipulations on patients with different degrees of disc degeneration as assessed by imaging to explore the biomechanical mechanisms of the two cervical spine rotation manipulations on the structures of the cervical spine under pathological conditions and at different degrees of degeneration. To make this model more useful for risk assessment of patients during manipulation.

## 5 Conclusion

A detailed finite element model of the whole cervical spine including the spinal nerve roots was developed and validated to simulate the key steps of two different cervical rotation manipulations to explore the biomechanical mechanisms of operation. The oblique pulling manipulation may be more suitable for the treatment of cervical spondylotic radiculopathy, while cervical rotation-traction manipulation is more suitable for the treatment of cervical cervical spondylosis.

## Data Availability

The raw data supporting the conclusion of this article will be made available by the authors, without undue reservation.
